# Perspectives of midwives on respectful maternity care

**DOI:** 10.1186/s12884-024-06894-1

**Published:** 2024-11-06

**Authors:** Petronella Lunda, Catharina Susanna Minnie, Welma Lubbe

**Affiliations:** 1 North-West University, Potchefstroom, South Africa; 2 University of the Western Cape, Bellville, South Africa

**Keywords:** Dignity, Humanizing care, Midwives, Nurses, Respectful maternity care, Woman-centered care

## Abstract

**Background:**

Respectful maternity care forms the foundation of maternity services; however, delivery of dignified, efficient, and effective care in these settings remains inconsistent. Research has identified several factors that influence respectful maternity care. To understand the South African context, these factors were explored and described from the perspectives of midwives.

**Methods:**

A qualitative descriptive inquiry was conducted, with participants recruited through snowball sampling on the social networking site Facebook. Semi-structured questions were used to collect data, to which participants responded in writing, detailing factors that influenced respectful care, including words and practices they associated with respectful maternity care. The data was analyzed using content analysis to identify common categories.

**Results:**

Twenty-five responses from participants were analyzed; four categories and six sub-categories emerged as representations of respectful maternity care. The categories and sub-categories were as follows: healthcare professional behavior (ethical conduct and professional attributes related to education and training), managerial support (conducive work environment), interpersonal facet of care (characteristics of healthcare providers), and the birth environment (caring within the birth environment and client-centered care).

**Conclusion:**

Midwives emphasized the importance of understanding respectful maternity care to ensure optimal outcomes for women, families, and communities. However, given the complexity of maternity care, it is crucial for policymakers, administrators, and midwives to comprehend and appreciate the various elements that define respectful care, as this understanding can significantly enhance its successful implementation.

## Introduction

Maternity care entails services provided to childbearing women from the prenatal period up to six weeks after childbirth [1, 2]. The services comprise antenatal/antepartum, intrapartum, and postpartum services of which respectful maternity care (RMC) is embedded within the care provided. The World Health Organization (WHO) [3] defines respectful maternity care as “care organized for and provided to all women in a manner that maintains their dignity, privacy, and confidentiality, ensures freedom from harm and mistreatment, and enables informed choice and continuous support during labor and childbirth”. During the different stages of a woman’s reproductive life, access to RMC is paramount. Women are at the center of maternity care services and deserve compassion and dignified care to enhance a positive ‘perinatal’ experience [4]. WHO [3] encourages facility births, but women often do not use them, even if available. Some reasons for avoiding healthcare facilities are neglect, long waiting periods, overcrowding, discriminatory care, and hurried care, to name a few [5–7]. Midwives mitigate these challenges by providing RMC which encourages the continued use of maternity care services crucial for improving maternal and neonatal outcomes.

## Background

Implementing and sustaining RMC requires a complete paradigm shift in individuals and the entire healthcare system since these contribute to respect or disrespect for women [8–10]. RMC is multidimensional, as many factors influence the provision of care. Factors identified include staffing levels, resources, collaboration among midwives, and skills [8, 10–12]. These factors can create challenging working conditions for midwives in maternity care settings. Thus, there is a need to make the working conditions conducive [11]. A study by Penfold et al. [12] explored “staff experiences of providing maternity services in Tanzania” and found that although midwives were resourceful in ensuring continued patient care, local authorities should still ensure that resources are adequate and broken equipment repaired or replaced promptly for optimal patient care. The authors concluded that inadequate equipment, drugs, and resources contribute to poor patient outcomes and demoralized midwives.

Inadequate staffing has been identified as a barrier to individualized care, as midwives are expected to attend to more than one woman at any time [9, 13]. Leaving a woman unattended for an emergency can result in adverse events should the woman develop a complication in the absence of a midwife [13, 14]. Inadequate staffing contributed to an increased workload that harmed the physical and psychological health of midwives. Midwives reported being exhausted, discouraged, and afraid of losing their practicing license in case of an adverse event [9]. Thus, in the maternity environment, midwives need support and collaboration from other healthcare professionals to render high-quality care [8].

However, successful collaboration requires mutual respect between professionals working in primary and secondary levels of care. Preceding collaboration is a positive engagement among midwives and a shared understanding related to protocols, guidelines, and models of care [15]. Additionally, to improve collaboration, training practices, and policies must be evaluated to align practices with RMC [8, 9, 11, 13]. To realize positive outcomes, midwives need continuous professional development, evidence-based practice (EBP), and implementation of a woman-centered care philosophy [16]. Authors Munabi-Babigumira et al. [16] concluded that communication and collaboration between professionals are vital in promoting efficient patient care. More so, acknowledging each other’s profession and understanding the different roles played will foster an enabling environment [17]. For midwives in remote areas, effective collaboration means a reliable communication and referral system to ensure continuity of care [8]. Since institutional practices influence the model of care and overall functionality of maternity care services, the practices should be evidence-based, and woman-centered care philosophy inclined.

The incorporation of evidence-based practices, a reduction in the medicalization of childbirth, and a women-centered philosophy are drivers of RMC [13, 16]. In their study, VanGompel et al. [13] found that cesarean delivery rates differed substantially from one institution to the other. Favorably, practices that promote vaginal birth and women’s choices reduced the rate of cesarean delivery. The authors concluded that the findings in the study reaffirm other study findings that endorse midwife-led care, which is non-medical, as it considers women’s uniqueness. A cultural change supported by all stakeholders through education is imperative [18]. Training practices, policies, and inter-professional collaborations to standardize maternity care and, subsequently, the provision of quality care need ongoing evaluation to ensure compliance with EBP [13]. For midwives in remote areas, effective collaboration means reliable communication and a proper referral system to ensure continuity of care [8]. Conclusively, collaboration between midwives and other healthcare professionals is imperative to address the knowledge gap [19].

The World Health Organization [3] endorses competency as essential for midwives to render safe and dignified care. According to a study by Daemers et al. [15] conducted in the Netherlands to explore factors influencing the clinical decision-making of midwives found that midwives’ knowledge is grounded in basic education and training, use of guidelines and protocols, in-service training, and continuous professional development, reading journals as well as consultation with colleagues. However, adherence to EBP depends on an individual midwife. A study by Bradley et al. [9] that investigated the impact of “too few staff and too many patients on obstetric care providers and the quality of care” in Malawi found that the care was compromised due to the high rate of turnover, which led to the utilization of non-permanent personnel, who were not ‘hands’ on. Non-permanent personnel lacked the skills and competencies to render high-quality maternity care. Additionally, midwives at the secondary level of care perceive those working in primary settings as lacking essential obstetric skills, hence the high referral rate [15]. Thus, training in Essential Steps in Managing Obstetric Emergencies (ESMOE) and a skills mix is vital. Oluoch-Aridi et al. [20] pointed out the importance of re-training midwives on soft skills to improve interpersonal relationships and complement clinical skills. Soft skills including good communication, friendliness, and empathy are desirable qualities of midwives [21, 22]. Importantly, desirable attributes help women relax and establish trusting relationships with midwives. Authors Khresheh et al. [23] concluded that besides personal attitudes, interaction with colleagues, and healthcare facility norms, EBP should influence midwifery decisions.

Literature has highlighted the complex factors that impact healthcare functionality. These factors are crucial in ensuring high-quality maternity care; thus, addressing them should be a priority.

### Design

A qualitative descriptive approach was used to capture participants’ perspectives on RMC using their own words [24, 25]. This method afforded data collection directly from individuals who represented the larger population.

### Methods

#### Population and sample

The population comprised midwives working in maternity settings in South Africa. The participants were selected by non-probability sampling using the snowball technique. A specific social networking site (SNS), Facebook, was chosen for recruitment as it provides extensive, timely, and cost-effective coverage. Facebook groups are cybernetic communities comprising people with common interests [26]. Relevant South African Facebook groups were selected with search words such as midwifery care, maternity care, and midwives. Permission was sought from group administrators.

An invitation was posted on closed Midwifery pages to recruit participants. Potential participants were invited to join the new Facebook page (Respectful Maternity Care), and a link directed them to the introduction page on the QuestionPro survey platform. The introduction page had information about the study and acted as the informed consent. Potential participants were requested to share the QuestionPro survey platform link with persons in their network who met the criteria but were not on Facebook (See Table 1). The sample for this study was 25. In exploratory research, a sample size of 10–30 is acceptable [27].

**Table 1:** Inclusion and exclusion criteria

Inclusion	Exclusion
- Midwives presently working in maternity settings in South Africa. - Midwives who are computer literate and can navigate the web and type in the responses.	- Non-practising midwives as they might not be up to date with current practices. - Agency staff - Midwives who are not fluent in English and may not clearly understand the questions.

### Data collection

The link to the QuestionPro survey platform took potential participants to an introduction page that represented informed consent. The introduction page explicitly highlighted that only a midwife was to complete the questionnaire to reflect authenticity. Information about anonymity and confidentiality, the voluntary nature of the study, expectations, and the freedom to withdraw from the study at any time by exiting the platform were shared. If the potential participant understood and accepted the outlined information and wanted to complete the survey, the next step was to click the **“next”** option to access the questionnaire. Thus, electronic informed consent was obtained from all the participants. No contact details were requested, and completed surveys were captured numerically according to the order of completion to safeguard anonymity.

Participants completed the questionnaire individually online. This ensured that the findings reflected the participants’ perspectives [28]. The online survey was open for four months from April-August 2021 to allow for maximum responses. The once-off response option was activated on the survey platform to allow only one response per participant. The researcher monitored the responses on the survey platform fortnightly, reported them to the supervisor, and reposted them on Facebook groups three times. The questionnaire was completed by 25 participants, with a response rate of 28.57%. The link was de-activated after four months. 

This study’s data collection comprised one close-ended and three open-ended questions (See Table 2). Open-ended questions allowed participants to reflect on the meaning of respectful care for them in detail, illuminating their perspectives [29]. (Table 2). Upon completing the survey, the participants were requested to click on the “send” option to have their responses recorded on the survey platform.

**Table 2:** The three open-ended questions

1. *List six factors that influence respectful maternity care for you.*
2. *List six words that represent respectful maternity care for you.*
3. *Write a short paragraph on maternity practices centered around respectful care in your facility.*

### Data analysis

The ATLAS.ti 8 computer program was used to analyze the data [30]. Data were imported from the survey platform onto a Microsoft Word document and into ATLAS.ti 8. Data analysis in qualitative studies consist of reading through the text to identify recurring phrases and organizing the findings according to similarities in meaning [31]. In this study the content analysis approach outlined by Elo and Kyngas was followed [32]. The process involved thoroughly reading the responses to identify similar and recurring phrases and words. These were then coded and organized based on their meanings, leading to the creation of categories and sub-categories. Data saturation was reached when no new concepts emerged. An independent co-coder also analyzed the data and a consensus meeting was held to compare and agree on categories to ensure the credibility of the findings [28]. Four categories with six sub-categories emerged. Each sub-category had elements that best described it as assigned.

### Trustworthiness

Trustworthiness ensures that the study findings are of good quality. This study confirmed trustworthiness by applying the four principles according to Lincoln and Guba [33]: credibility, transferability, dependability, and confirmability. Credibility was assured by a thorough, prolonged engagement attained by leaving the survey open for four months. Transferability was reinforced by describing the research setting and findings based on participants’ responses. Dependability, on the other hand, was enhanced through descriptions of the methodological process of the study from conception to finalization. Further strengthening of the data was done with an independent co-coder. This ensured that there was no bias [34]. Confirmability in this study was achieved through reflexivity and supported by raw data.

## Results

Twenty-five midwives completed the questionnaire.

- **Characteristics of midwives**.

The participants were midwives with at least two years of experience in maternity settings and were actively practicing in these environments. All participants were aged 18 years and older. This study focused on understanding midwives’ perspectives of respectful care, without examining other factors. Consequently, gender, years of experience, and age were not included in the demographic analysis.

According to participants’ perceptions, respectful maternity care is a multi-faceted approach shaped by numerous factors. Direct quotations, labeled with participant numbers, were used to capture the authentic views of the midwives. Four categories emerged from these perceptions: healthcare provider behavior, managerial support, interpersonal aspects of care, and the birth environment (See Table 3).

**Table 3:** Categories and sub-categories related to perspectives of respectful maternity care

CATEGORY 1 Healthcare provider behavior	CATEGORY 2 Managerial support	CATEGORY 3 The interpersonal facet of care	CATEGORY 4 The birth environment
Sub-category 1: Ethical Conduct	**Sub-category 1**: Conducive work environment	**Sub-category 1**: Characteristics of healthcare providers	**Sub-category 1**: Caring within the birth environment
Sub-category 2: Professional attributes related to education and training			**Sub-category 2**: Client-centered care

### CATEGORY 1: Healthcare provider behavior

This category highlights sub-categories as factors associated with the provision of RMC: the ethical conduct of the midwife and attributes related to education and training.

### Sub-category 1: Ethical conduct

According to participants, ethical conduct represented acceptable behavior aligned with the profession’s code of conduct. Participants described ethical conduct as an important aspect in the provision of non-discriminatory, efficient, and dignified care.

- **The meaning of respect**,** according to the participants**.

Respect was a recurring concept among participants who equated it to ethical conduct. The midwife’s behavior should be acceptable and conform to the professional ethical code of conduct. For participants, respect signified equal treatment of women, maintaining confidentiality, and providing feedback, confidentiality, and feedback.

At our “Mother and Baby Clinic,” every woman is treated with love and dignity, and all women are treated equally, no matter their social, economic, or racial status. (P18)

Partnership with women to promote self-care and the health of unborn babies and families. (P 16)

…no patient should feel unattended or worthless during any interaction. (P21)

Confidentiality during history taking, examination, and feedback sessions, e.g., HIV g or genetic counselling. (P21)

### Sub-category 2: Professional attributes related to education and training

Education and training are the cornerstone of the midwife. Thus, professional attributes pertain to knowledge and skills representing competence. A competent midwife has met all the training requirements and continues improving their knowledge and skills.

### Knowledge and skills

The participants pointed out that competence stems from training and that knowledge and skills are integral to competence. Concepts such as *assessment*,* investigations*,* and referral* are part of the competencies needed to provide optimal care to women.

The care workers conduct drills and in-service training to keep sharpening their skills and knowledge, women are assisted in making a birth plan, and their beliefs and expectations are considered, but the women are, however, given information on the care being provided to them and are made to understand at their level that certain laws need to be followed. (P01).

According to participants, identifying risks and referring women to a higher level of care depended on the ability to assess and diagnose. Participants acknowledged continuous in-service training on the latest evidence and guidelines.

### CATEGORY 2: Managerial support

Managerial support is crucial in creating an enabling environment for midwives to provide high-quality care. Access to well-functioning equipment, adequate staffing, and appropriate infrastructure directly influences midwives’ ability to deliver safe, timely, and respectful care. When these elements are in place, midwives can focus on their responsibilities while enhancing patient outcomes and job satisfaction. The support reinforces midwives’ role as caregivers and advocates, promoting an environment where they can operate effectively and uphold ethical standards. Participants cited these factors as antecedents to the provision of optimal maternity care.

### Sub-category 1: Conducive work environment

The support cited by participants was adequate human resources, infrastructure, equipment, and medical supplies. A conducive environment entails sufficient human resources, equipment, medical supplies, and appropriate infrastructure. Participants used phrases such as “poorly equipped, overburdened, and burnout” to describe a non-conducive work environment.

- **Human resources**.

Human resources refer to midwives. Participants reflected a need for adequate midwives who can render care effectively. Participants described human resource shortage using phrases such as “availability, skilled healthcare providers, understaffing, overburdened.”

South African maternity facilities are understaffed, poorly equipped, and overburdened. Training more midwives and assistants to educate pregnant women about pregnancy and labor, attendance of antenatal care of women early with a supportive person or doula, and well-prepared women for postpartum care and the baby will help ease the burden on the under-resourced maternity facility can improve women’s satisfaction. (P10)

Support from managers, human resources that is adequate staffing, the patient-to-midwife ratio, the morale of staff healthcare workers…adequate equipment that facilitates work being done efficiently. (P15)

Lack of support from management affected staff morale leading to burnout, bureaucratic leadership, and burnout. (P25)

According to participants in this study, adequate human resources enable them to fulfill their roles efficiently as a high workload contributes to burnout from fatigue.

- **Equipment and medical supplies**.

Participants acknowledged the importance of adequate equipment and medical supplies in enabling respectful care. Common aspects mentioned by participants were functioning equipment and sufficient stock and the coordination by relevant stakeholders to ensure equal distribution of medical supplies.

Adequate equipment that facilitates work. (P15)

Enough utilities, stock, resources, and functioning equipment. (P10)

When supplies are out, the sharing of the stock between facilities is coordinated by the sub-district offices. Every stakeholder is engaged. (P23)

Adequate equipment and medical supplies facilitated the provision of patient’s needs. The consequence of inadequate medical supplies or equipment was compromised patient care.

- **Infrastructure**.

Infrastructure refers to the physical space available to accommodate women without overcrowding. According to participants, the infrastructure was inadequate to accommodate women seeking maternity care services and their support persons. Participants used phrases such as *“challenges and*,* overcrowding”* to describe the state of the infrastructure.

Infrastructure is limited to allow doulas. (P13)

Ward capacity versus the area for providing care […] the number of women we are providing care is increasing monthly. (P14)

Overcrowding at government hospitals. (P19)

### CATEGORY 3: Interpersonal facet of care

This category describes interpersonal relationships during engagement between the women and the midwives. Most importantly, how midwives relate to or treat women contributes to respectful care. Participants perceived interpersonal relationships as a product of a positive engagement with women and compassion from the midwife.

### **Sub-category 1: Characteristics of midwives**

An approachable demeanor by midwives can enable women to relax as described by participants in this study. These characteristics are empathy, love, passionate care, and effective communication. A “smile, gentleness, warmth, non-judgmental attitude, paying attention and calling a patient by name” represented good attributes.

Being there and available to the mother during labor. Understanding her perception of pain and addressing possible and potential challenges…Calling the patients by their preferred names. (P09)

Being kind and considerate. Therefore, I am committed to providing midwife-led maternity care that contributes to the optimal health of low-risk pregnant women, newborns, and families. (P08)

Women are welcomed with a smile upon admission to the labor ward. Being orientated on the ward and routine, more importantly, on what to expect during the phases of labor. (P05)

Participants highlighted that a caring attitude and compassion towards women were critical in eliciting trust and confidence from women. Participants believed that the type of engagement with women subsequently influenced women’s perceptions of care.

### Sub-category 2: Effective communication

Communication enables the sharing of information between the parties involved. However, the information should be simple and clear for the recipient to comprehend. Participants reflected that addressing women by name, listening to them, and giving relevant advice was vital. Communication should be in *“a language understood by the woman.”*

Addressing women in their languages. Involve them in the treatment plan. Having to make informed decisions about their care plan and treatment. (P03)

I believe giving information to all women, about their conditions, the options they have, and explaining procedures beforehand irrespective of their social status, and allowing them the freedom to give informed consent ensures cooperation, is acceptable to all and will give women a sense of worth. (P08)

Self-introduction to patients. Calling the mother by the name she prefers. Allowing her to make informed choices and have a listening ear to her, not giving false reassurance asking for permission to do procedures on the woman and giving her the report of the findings. Allowing her freedom of speech and movement if there are no complications. (P11)

On booking ANC, a woman is assigned to a primary healthcare ward-based outreach team member to seek clarity on aspects untouched or not yet counselled on. All necessary explained investigations are carried out by consent, verbal or written. (P23)

Communication is an ongoing process and should be in a language understood by the woman using simple terms to make informed decisions.

### CATEGORY 4: The birth environment

The birth environment should be safe and comfortable for the women to feel safe and cared for. Hence, maternity care practices need to support women. This category is discussed under the two sub-categories of caring within the birth environment and client-centered care.

### Sub-category 1: Caring within the birth environment

A caring birth environment should be supportive, and family-friendly for women.

According to participants, the characteristics of a caring birth environment are a non-medicalized environment that provides a ‘safe space’ for women under care, offers privacy for women, and allows free mobility for them.

- **Supportive environment**.

According to participants, a supportive environment is a safe space for women; it allows free mobility and provides a milieu conducive to childbirth. Phrases such as *“warm and safe and quiet*,* well-lit and pain management*,* the ability to ambulate during labor”* described a safe and supportive environment.

A supportive environment includes emotional and physical support for women. Participants acknowledged the importance of creating a therapeutic environment for women.

- **Family-friendly environment**.

A family-friendly environment accommodates the woman’s family and recognizes them as primary support. A family-friendly environment was described using phrases such as *“doula*,* include families*,* birth companion.”* However, the COVID-19 pandemic changed the landscape of ‘birth companionship’ as narrated by some participants. *“Before COVID-19*,* we always encouraged the women to have a birth companion*”.

Women and families are always given information about their conditions, family involvement is encouraged, and where women wish to include their families, families are given information; participation of both the woman and the family is always encouraged. (P08)

Women can have their doulas around for comfort and a sense of home amidst the COVID-19 pandemic; the midwife no longer just delivers or monitors the woman in labor but has become family to the mothers, as they have no family permitted to visit during these times. (P03)

Before COVID-19, we allowed every pregnant woman in labor to have a doula, be it a partner or relative, for support during labor, but due to COVID-19, that is no longer the case. With COVID-19 being the new normal, we still provide privacy and respectful care. (P14)

Participants acknowledged the importance of birth companionship. However, most of the data was collected during the COVID-19 pandemic; thus, the COVID-19 pandemic led to some restrictions, especially related to visitation, to mitigate transmission of the virus.

### Sub-category 2: Client-centered care

A client-centered approach respects women’s choices, maintains dignity for women, and does not discriminate. Participants acknowledged that women’s preferences are central to their care. A client-centered care focuses on women’s right to choose. This sub-category was discussed under the following elements: respect for women’s choices, upholding dignity for women, and culturally sensitive care.

- **Respect for the choices of women**.

Women have varying wishes and needs. Participants acknowledged the importance of accommodating women’s unique needs and wishes. Phrases such as *“allow women to choose*,* enema and episiotomy are not given routinely*,* but rather as options that the women have”* described how women’s choices were accommodated.

Our facility is a regional hospital, but we always allow women to give birth in our facility even though they are not high-risk because we believe allowing women to give birth in their preferred facility is likely to reduce fears and inspire cooperation and trust… Offering beneficial procedures in a non-coercive manner, respecting the woman’s choice. (P08)

Women should be encouraged to deliver in comfortable positions and allow labor to progress naturally. (P10)

Participants pointed out their role in ‘working’ with women and assisting them in making healthy, and informed choices. In doing so, women felt empowered and valued.

- **Upholding the dignity of women**.

According to participants, women need to give birth in a dignified environment. A dignified environment meant listening to women, providing privacy, and advocating for women. Women also had *“Freedom of speech and movement if no complications.”* For the participants, privacy entails eliminating unnecessary exposure of women during examination by using an individual room or space while safeguarding women’s information through confidentiality.

Our clients are treated respectfully, and… delivered in private/individual rooms. (P03)

Respect for human dignity and women as persons with full human rights. Advocacy for women’s voices to be heard, and they can make autonomous decisions. (P16)

It was clear that upholding dignity for women entails equal treatment for all women. All women have the right to access maternity care services without being discriminated against. Ensuring privacy and autonomy contributes to dignified care.

- **Culture-sensitive care**.

Cultural sensitivity refers to sensitivity to different belief systems, religions, traditions, and practices. The care is non-judgemental. Participants acknowledged that culture-sensitive care forms part of the care provided to women through phrases such as *“respect all women irrespective of age*,* race*,* social status or religion”.*

Making her feel accepted despite the differences of religion, culture, creed, nationality, race, and social standing. (P09)

Advocate for women so that their voices are heard, they can make autonomous decisions, and that healthcare providers’ cultural practices do not harm women’s and babies’ health and focus on health promotion and disease prevention. (P16)

Respect for personal views and beliefs is an aspect that supports cultural sensitivity. Participants acknowledged the importance of culturally sensitive care for women as part of respectful care.

## Discussion of findings

This study explored respectful maternity care from the perspectives of midwives. Participants described respectful maternity care as comprehensive care rendered to women during every encounter. The care consists of various actions such as empathy, sensitivity, compassion, and caring. Other aspects included respect for a woman’s values and beliefs and paying attention to a woman’s needs in a humane way through acts of kindness. These soft skills are important key components of respectful maternity care as they contribute to compassionate care, and dignified. The four categories along with their respective sub-categories will be the basis of the discussion; healthcare provider behavior, managerial support,  the interpersonal facet of care, and the birth environment.

### Healthcare provider behavior

Ethical conduct and competency form the backbone of midwifery care, influencing the quality of care and rapport between midwives and clients. When midwives follow an ethically sound framework, they’re better equipped to deliver respectful, culturally sensitive, and patient-centered care.

- **Ethical conduct**.

The participants in this study described ethical conduct as an essential aspect of providing respectful maternity care. According to participants, ethical conduct entails acceptable behavior that conforms to the professional ethical code of conduct. Thus, respect meant partnership, equal treatment, privacy, confidentiality, and feedback. Similarly, Rodrigues et al. [35] highlighted that ethical conduct is an essential part of caring to propel empathy, compassion, and gentleness. In agreement, Dippenaar and da Serra [36] state that midwives should display a caring attitude, courteousness, competence, and professionalism to expedite trusting interpersonal relationships with women. Thus, ethical conduct is a crucial element of respectful care.

Respectful maternity care should include appropriate care according to a woman’s needs [37]. Midwives should ethically conduct themselves by following the professional code of conduct and philosophy (South Africa Nursing Act 33 of 2005) [38]. Ethical conduct is the cornerstone for providing RMC; hence, midwives should respect and treat women as partners in care and not just passive recipients of care. The International Confederation of Midwives (ICM) [39] emphasizes that midwives should display behavior that upholds the profession’s standards by collaborating with women.

- **Professional attributes related to education and training**.

According to participants in this study, competence meant having knowledge, clinical skills, and the ability to diagnose and refer the woman to avert complications. Participants regarded competence as a product of continuous education. Likewise, Hunter et al. [40] underlined that professional training is the foundation for knowledge, skills, and attributes as it affects the care rendered to women. Orpin et al. [37] recommend that respectful care be part of basic education and continuous in-service training to keep midwives compliant with EBP. From the participants and literature, education and training are the foundation for knowledge and skills that render safe and efficient care.

### Managerial support

Managerial support is crucial in creating an enabling environment for midwives to provide high-quality care. Access to well-functioning equipment, adequate staffing, and appropriate infrastructure directly influences midwives’ ability to deliver safe, timely, and respectful care. When these elements are in place, midwives can better focus on their responsibilities while enhancing patient outcomes and job satisfaction. The support reinforces midwives' role as caregivers and advocates, promoting a setting where they can operate effectively and uphold ethical standards.

- **Conducive work environment**.

Participants in this study highlighted the need for adequate staff to prevent a high workload that leads to burnout. Similarly, Hunter et al. [40] highlighted that limited resources in maternity care settings contribute to trends that harm the provision of RMC. A study entitled “Too few staff, too many patients” by Bradley et al. [9] revealed that midwives complained about stress-related high workloads and were contemplating resigning. In agreement, Ndwiga et al. [10] state that staff shortage and high workload lead to burnout and compromised care. Hence, managers should address staffing norms. Similarly, authors Dalinjong et al. [41] and Okedo-Alex et al. [42] found that inadequate equipment and medical supplies compromised maternity care. Concurringly, a study by Moyimane et al. [43] revealed that poorly maintained equipment, inadequate medical equipment and supplies compromised patient care.

Participants in this study pointed out that dilapidated and inadequate physical infrastructure leads to overcrowding. Other studies by Okedo-Alex et al. [42], Shimpuku et al. [44], and Warren et al. [45] highlighted that limited space compromises privacy and confidentiality. Thus, to promote respectful care, the physical infrastructure should be well maintained and adequate to accommodate women and prevent overcrowding [46, 47].

Physical infrastructure, inadequate equipment, and medical supplies challenges are complex issues that require addressing within maternity settings in the local context to ensure that care is not compromised.

### The interpersonal facet of care

Interpersonal relationships are central to the caring dynamic between midwives and women. Qualities such as empathy, respect, active listening, and open communication play a significant role in fostering trust and comfort. When midwives display gentleness, attentiveness, and respect for individual preferences, they’re more likely to build rapport and encourage women to engage openly in their care.

- **Characteristics of Midwives**.

Participants in this study described desirable characteristics as empathy, love, care, and effective communication with women. The afore-mentioned characteristics represent compassionate care. Desirable attributes are smiles, gentleness and empathy, attentiveness, and addressing a patient by name. Oluoch-Aridi et al. [20] pointed out the importance of re-training midwives on soft skills to improve interpersonal relationships and complement clinical skills. Concurringly, good communication skills, friendliness, and empathy are desirable qualities of midwives [21, 22]. Desirable attributes help women relax and establish a trusting relationship with midwives. Authors Khresheh et al. [23] concluded that engaging them with these attributes would elicit confidence and expedite good interpersonal relations with midwives.

Furthermore, a study by Krause et al. [48] revealed that midwives used continuous communication to provide informational support to women through advice, guidance, and feedback. Favorably, good communication contributes to good interpersonal relationships. Chadwick et al. [49] point out that establishing good interpersonal relationships with women is vital in humanizing maternity care. Conclusively, Gibson et al. [50] stated that women have the right to information related to their care and available options to make informed decisions. In this study, participants highlighted that communicating in a woman’s language was an effective medium. Burrowes et al. [5] concur with this statement that an unfamiliar language can be a barrier to effective communication. More so the ICM [39] recommends that communication should be in a language understood by the women. If not familiar with the woman’s language, an interpreter is encouraged.

Effective communication fosters strong interpersonal relationships. Midwives should understand that their personal attributes and communication style can signifi­cantly influence their relationships with women, either positively or negatively.

### The birth environment

Midwives can create and maintain a safe and comfortable birthing environment for women through practices that acknowledge and support women. Participants in this study pointed out that caring within the birth environ­ment and client-centered care is vital in creating a space where women feel safe and acknowledged.

- **Caring within the birth environment**.

According to participants in this study, the features of a caring birth environment are a non-medicalized environment, privacy, freedom of speech, and mobility for women. Limited space and overcrowding were aspects that participants cited as contributing to the lack of privacy. Concurringly, Berg et al. [51] reaffirm that women need a supportive environment to feel dignified, secure, and at ease. However, sometimes overcrowding within the birth environment results in limited mobil­ity and lack of privacy, compromising the dignity of women [37].

According to the study’s findings, a family-friendly environment recognizes and accommodates the woman’s family as the source of primary support. Back in the early 1970s, a study by Enkin [52], titled “Family-Centred Maternity Care”, documented that “instead of fostering husband-wife communication and maturation, our patterning of obstetrical care tends to produce separation and regression.” However, midwives, women, and families need a shared view of pregnancy and childbirth [40]. Hence, midwives should involve women and family members in maternity care-related issues throughout pregnancy and childbirth. However, participants in this study reflected that, due to the COVID-19 pandemic, there were restrictions on visitation to mitigate the spread of COVID-19. Authors Pavlidis et al. [53] highlighted the importance of EBP, to ensure a safe birth environment. However, the birth environment should be accommodative towards women and their families to foster a milieu conducive to childbirth.

- ***Client-centered care***.

This study revealed that midwives acknowledged women as crucial in that client-centered care respects women’s choices, maintains their dignity, includes non-discriminatory care, and upholds dignity for women through culturally sensitive care. Hunter et al. [40] and De Lubrusse et al. [54] acknowledge cultural-sensitive care when pointing out that person-centered maternity care focuses on women’s needs and preferences in line with their values and belief systems. Client-centered or woman-centered care is a philosophy that recognizes patients’ needs as the center of care. Downe et al. [55] reaffirm that maternity care should acknowledge personal views, belief systems, and traditional practices, as these aspects matter to women. Midwives should, therefore, provide care integrated with the traditions and values of women as part of client-centered care.

Culture-sensitive care is context-bound; thus, midwives should be aware of cultural practices’ diverse nature within their communities [36]. Women should not be discriminated against due to their beliefs or practices but supported to make healthy choices. Hunter et al. [40] complement that women-centered care requires that all midwives have a ‘buy-in’ of the philosophy to be successful. The role of the midwives includes supporting women in making informed choices so that they exercise autonomy [54, 56]. In agreement, midwives in a study by Moyer et al. [57] validated that informed consent was necessary when rendering care to women. Krause et al. [48] support the previous statements when stating that women’s autonomy in making informed decisions based on alternative options was imperative regarding preferences. Thus, a client-centered approach treats women holistically as individuals with varying backgrounds, needs, beliefs, and preferences.

## Conclusion

Addressing the factors influencing respectful perinatal care is critical for preventing compromised care during the perinatal period. These factors can either accelerate or hinder the delivery of respectful care. Therefore, addressing them could significantly enhance the quality of perinatal care provided. Respectful maternity care is fundamental in providing high-quality services encouraging women to appreciate and continue utilizing healthcare facilities. This study highlights midwives’ perspectives on respectful care, revealing a deep understanding of the fundamental elements contributing to safe, compassionate, and high-quality maternity care. Key components identified include ethical conduct, professional education, a supportive work environment, and managerial support. The emphasis on these factors, alongside a conducive birthing environment and woman-centered care, underscores midwives’ recognition of the broader systemic support necessary to sustain respectful maternity practices.

Moreover, midwives’ dedication to client-centered care and strong interpersonal relationships underscores their commitment to honoring each woman’s dignity and individuality. These insights affirm midwives’ pivotal role in promoting respectful maternity care and highlight the importance of a coordinated, system-wide approach to support this goal. Addressing the identified components and fostering a culture of respect and compassion within healthcare settings can greatly enhance care quality, ensuring that it is responsive to the needs of women, their families, and communities.

A limitation of this study is the omission of participant characteristics, such as age, years of service, and gender, which could provide additional insights into how these factors shape midwives’ perspectives. It is recommended that future research include these variables, offering a more nuanced and comprehensive understanding of respectful maternity care.

## Data Availability

Data sharing is available on request. Contact the corresponding author to request data and materials.

## Abbreviations

D&A: Disrespect and Abuse
EBP: Evidence-Based Practice
ESMOE: Essential Steps in Managing Obstetric Emergencies
ICM: International Confederation of Midwives
RMC: Respectful Maternity Care
WHO: World Health Organization
